# Monoacetyldiacylglycerols as emerging immunomodulatory lipids in chronic inflammation: mechanisms, biology, and translational potential

**DOI:** 10.3389/fimmu.2026.1927836

**Published:** 2026-10-07

**Authors:** Charles F. Manful, Dasinaa Subramaniam, Eric Fordjour, Lord Abbey, Raymond Thomas

**Affiliations:** 1 School of Science and the Environment, Memorial University of Newfoundland, Corner Brook, NL, Canada; 2 Biotron Experimental Climate Change Research Centre/Department of Biology, University of Western Ontario, London, ON, Canada; 3 Department of Plant, Food and Environmental Sciences, Dalhousie University, Truro, NS, Canada

**Keywords:** immunomodulation, lipid mediators, macrophage polarization, neutrophils, NF-κB pathway, STAT3/STAT6 signaling

## Abstract

Chronic inflammatory diseases remain a major global health burden, with approximately 30–40% of patients responding inadequately to current cytokine-targeted biologics, Janus kinase (JAK) inhibitors, or small-molecule immunomodulators. Beyond suboptimal efficacy, these therapies carry well-documented adverse effects, including infection and malignancy risks associated with JAK inhibitors, and act on single molecular targets, leaving upstream, membrane-proximal drivers of chronic inflammation largely unaddressed. Monoacetyldiacylglycerols (MacDGs), a structurally distinct class of partially acetylated triglycerides, have emerged as promising immunomodulatory lipids that act concurrently across multiple inflammatory signaling nodes, including STAT3/STAT6 and NF-κB pathways, macrophage polarization, and epithelial barrier integrity, offering mechanistic breadth that single-target therapies do not provide. Current evidence, derived predominantly from the synthetic analog palmitic–linoleic–acetyldiacylglycero (PLAG), demonstrates reproducible suppression of signal transducer and activator of transcription 3/6 (STAT3/STAT6) and nuclear factor kappa-light-chain-enhancer of activated B cells (NF-κB) signaling, rebalancing of Th17/regulatory T cell (Treg) dynamics, context-dependent control of neutrophil activity, and stabilization of epithelial barriers under inflammatory stress across *in vitro* systems, *in vivo* models, and early clinical studies. By integrating these convergent effects across immune and epithelial compartments, this review frames MacDGs as a coherent class of membrane-proximal immunomodulatory lipids. Despite these advances, major uncertainties remain regarding endogenous biosynthesis, molecular targets, pharmacokinetics, metabolic fate, and structure–activity relationships. As the first integrated synthesis to frame MacDGs as a coherent immunomodulatory lipid class rather than isolated experimental agents, this review consolidates foundational chemistry, biological occurrence, mechanistic insights, therapeutic applications, and translational challenges, offering a critical assessment of both the promise and substantial uncertainties that define this emerging field.

## 1 Introduction

Chronic inflammatory diseases, including rheumatoid arthritis, inflammatory bowel disease, asthma, acute lung injury, and metabolic inflammation, remain therapeutically challenging despite advances in cytokine-targeted biologics and JAK inhibitors (1). Approximately one-third of patients fail to achieve adequate disease control (2, 3), highlighting the need for agents that intervene at upstream, multilayered nodes of inflammatory regulation.

Chronic inflammation involves dysregulated neutrophil trafficking, maladaptive macrophage polarization, impaired epithelial barriers, and persistent membrane-proximal signaling, in addition to classical cytokine networks (4, 5). This complexity has driven interest in bioactive lipids that modulate inflammation at the interface of membrane organization, immune signaling, and epithelial defense. Within this expanding lipid landscape, monoacetyldiacylglycerols (MacDGs) have emerged as structurally distinctive molecules with actions spanning multiple immune and epithelial compartments (6, 7).

Two classes of bioactive lipid mediators have been most extensively characterized in the context of inflammatory regulation. Eicosanoids, including prostaglandins, leukotrienes, and thromboxanes, are generated via cyclooxygenase (COX) and lipoxygenase (LOX) enzymatic conversion of arachidonic acid and act through discrete G protein-coupled receptors to modulate vascular permeability, leukocyte recruitment, and pain sensitization, predominantly amplifying acute inflammatory responses (8). Specialized pro-resolving mediators (SPMs), including lipoxins, resolvins, protectins, and maresins, arise from enzymatic conversion of polyunsaturated fatty acids (9, 10) and act through specific receptors (e.g., ALX/FPR2, DRV1/GPR32) (11) to actively promote inflammation resolution, efferocytosis, and tissue repair without broadly suppressing immune defense (12). Both classes are defined by enzyme-specific biosynthesis and receptor-mediated signaling, conferring pharmacological precision but also inherent specificity that limits their action to defined pathways. MacDGs are mechanistically distinct from both: no COX-, LOX-, or receptor-dependent biosynthetic or signaling pathway has been established for this class, and current evidence suggests they act through broader modulation of membrane organization and multiple parallel transcriptional axes rather than through a single enzyme-substrate-receptor cascade. Notably, MacDGs have demonstrated early clinical activity in inflammatory contexts where eicosanoid-targeted therapies have shown limited efficacy (discussed in Section 5), suggesting potential complementarity rather than redundancy. Whether MacDGs represent a complementary upstream regulatory layer to eicosanoid and SPM biology, or an entirely independent immunomodulatory mechanism, remains an open question that this review addresses.

MacDGs are partially acetylated triglycerides defined by a sn-3 acetyl group and diverse sn-1/sn-2 fatty acid combinations (13). Most mechanistic understanding derives from the synthetic analog palmitic–linoleic–acetyldiacylglycerol (PLAG also known as EC-18), which demonstrates reproducible suppression of STAT3/STAT6 and NF-κB signaling, modulation of innate immune responses, and stabilization of epithelial barriers under inflammatory stress (14, 15). These pleiotropic actions suggest MacDGs influence inflammation at membrane-proximal checkpoints rather than through single receptors, providing preliminary mechanistic rationale for evaluating MacDGs in disorders where innate immune amplification and epithelial dysfunction drive chronic pathology, though primary molecular targets remain unidentified (16–18).

Clinical translation has begun, with completed Phase 2 validation in chemoradiation-induced oral mucositis demonstrating reduced severity and duration (19), pilot evidence of reduced neutropenia incidence (20, 21), and ongoing Phase 2 evaluation in moderate-to-severe atopic dermatitis (NCT06487000). These early clinical findings provide proof-of-concept for MacDG activity in human inflammatory disease while highlighting critical gaps in dose optimization, patient selection, and comparative effectiveness that require resolution.

Despite these promising attributes, the field remains defined by unresolved questions regarding endogenous biosynthesis, primary molecular targets, metabolic fate, and structure–activity relationships. This review synthesizes current knowledge on MacDG structure, biological occurrence, mechanistic activity, therapeutic potential, and translational barriers, offering an integrated framework for understanding the emerging role of MacDGs in chronic inflammatory disease while critically examining the substantial uncertainties that must be addressed before credible therapeutic advancement.

## 2 Chemistry and structural features of monoacetyldiacylglycerols

### 2.1 Molecular architecture and biophysical implications

Monoacetyldiacylglycerols are glycerolipids characterized by esterification of long-chain fatty acids at the sn-1 and sn-2 positions and a short acetyl moiety at sn-3 (22). This substitution increases polarity relative to classical triacylglycerols, weakens hydrophobic packing, and is predicted to increase lateral mobility within lipid assemblies. Biophysical modeling suggests that the acetyl group alters the glycerol backbone orientation and may preferentially localize MacDGs to less ordered membrane microdomains (23, 24), though direct experimental evidence for MacDG membrane partitioning dynamics, diffusion coefficients, or microdomain localization remains to be established through super-resolution imaging or fluorescence recovery techniques. These predicted features imply that MacDGs could influence the physicochemical environment encountered by transmembrane receptors and membrane-proximal signaling assemblies (25, 26). In the literature, these compounds have also been described as monoacetyldiglycerides; however, to emphasize their triester glycerol backbone and to maintain consistency with contemporary lipid nomenclature, we use the term monoacetyldiacylglycerols (MacDGs) throughout this review.

Despite superficial resemblance to diacylglycerols (DAGs) (**Figure 1**), MacDGs show no canonical DAG-dependent signaling via C1-domain proteins like PKC (27). Acetyl substitution appears to disrupt lipid–protein interactions essential for DAG biology, distinguishing MacDGs as a functionally distinct class.

**Figure 1:**
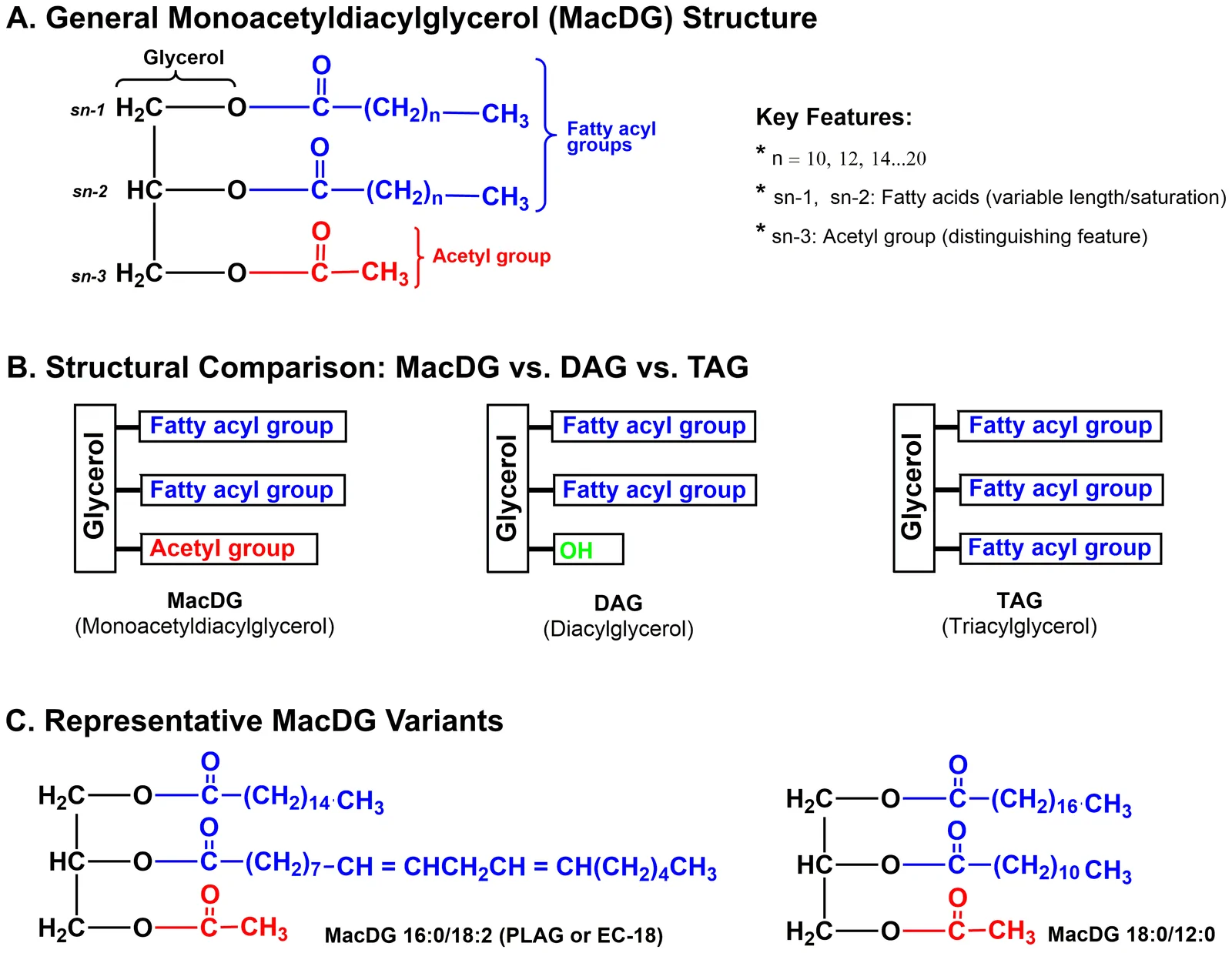
Structural comparison of monoacetyldiacylglycerols (MacDGs) and related glycerolipids. **(A)** General structure showing sn-1 and sn-2 long-chain fatty acids and sn-3 acetyl group. **(B)** Comparison with diacylglycerol (DAG) and triacylglycerol (TAG). **(C)** Representative natural MacDG variants detected in biological samples.

### 2.2 Structural diversity and the PLAG-centric evidence base

Natural MacDG variants, including saturated, monounsaturated, and polyunsaturated species, span chain lengths from C12 to C22 (28, 29). Trace polyunsaturated MacDGs containing eicosapentaenoic acid or docosahexaenoic acid have been detected in marine-derived oils and select mammalian tissues (30). However, predominance of synthetic PLAG in modern research has limited systematic exploration of structure–activity relationships. Whether the immunomodulatory properties attributed to PLAG reflect class-wide phenomena or unique behavior of this particular analog remains unknown. Systematic structure–activity relationship mapping across chain length, saturation, stereochemistry, and positional isomerism is therefore essential for rational therapeutic development.

### 2.3 Biosynthesis and endogenous origins

No specific mammalian acyltransferase, esterase, or other enzyme directly catalyzing sn-3 acetylation of diacylglycerol has been identified, and no defined endogenous biosynthetic pathway has been established for MacDGs in mammals (28, 31). Proposed associations between MacDG occurrence and physiological states such as stress, infection, or metabolic perturbation are based on indirect observational evidence from detection studies rather than direct mechanistic investigation (discussed further in Section 3.3); these should therefore be regarded as hypothesis-generating rather than established biological relationships. Plausible alternative or contributing sources of tissue MacDG content, including dietary or exogenous exposure, microbiome-associated production, incidental generation during neutral lipid remodeling, or analytical misannotation of closely related lipid species, cannot currently be excluded.

### 2.4 Gastrointestinal fate, cellular uptake, and metabolic processing

The gastrointestinal fate of MacDGs following oral ingestion remains incompletely characterized, representing a significant knowledge gap with direct implications for dose selection, formulation strategy, and interpretation of systemic pharmacological effects. Based on general acylglycerol digestion principles, MacDGs would be expected to encounter luminal lipolysis by lingual lipase and pancreatic lipase, both of which preferentially hydrolyse ester bonds at the sn-1 and sn-3 positions of glycerolipids (32, 33). However, the sn-3 acetyl group of MacDGs is structurally atypical relative to conventional long-chain fatty acid esters at this position; whether luminal lipases and esterases hydrolyse this short-chain acetate moiety with the same efficiency, more rapidly, or more slowly than conventional sn-3 long-chain esters has not been directly investigated for PLAG or any MacDG species. If rapid sn-3 hydrolysis occurs, intact MacDG absorption would be unlikely and the biologically active species may be the resulting atypical diacylglycerol rather than the parent compound. If the acetyl group confers esterase resistance, intact MacDG may reach enterocytes and be absorbed via fatty acid-binding protein-mediated pathways analogous to conventional diacylglycerols (32). The limited oral bioavailability of native PLAG (34, 35) is consistent with either scenario and does not distinguish between them.

Cellular uptake and intracellular metabolic fate are similarly unresolved. Whether MacDGs are incorporated into chylomicrons and transported via the lymphatic system, enter portal circulation as free fatty acids following complete hydrolysis, or accumulate in enterocytes as atypical DAG intermediates is unknown. Should hydrolysis products reach the enterocyte cytoplasm, free fatty acids released from sn-1 and sn-2 positions would be expected to enter conventional TAG resynthesis via the monoacylglycerol or glycerol-3-phosphate pathway (32, 33); the fate of the acetate moiety released from sn-3 hydrolysis is less clear, as short-chain acetate may enter mitochondrial acetyl-CoA metabolism rather than lipid resynthesis pathways, though this has not been directly examined for MacDG-derived acetate specifically. Cell-type differences in lipase expression, fatty acid-binding protein isoforms, and intracellular lipid trafficking machinery suggest that MacDG digestion, uptake, and intracellular processing are likely to vary between enterocytes, immune cells of the intestinal lamina propria, and hepatocytes receiving portal-delivered lipid metabolites; whether such variation translates into meaningful differences in MacDG bioactivity across these compartments has not been investigated.

The potential contribution of gut microbiota to MacDG metabolism warrants consideration. Microbial lipases and esterases are known to generate MacDGs in fermented food matrices through acylation and esterification reactions (Section 3.1), and gut bacterial phospholipase and esterase activities are documented to produce diacylglycerol species in the human intestine with consequences for host lipid metabolism that remain incompletely understood (36). Whether resident intestinal microbiota similarly metabolize, biotransform, or generate MacDGs from dietary precursors *in vivo* has not been investigated. Advanced delivery systems such as solid self-nanoemulsifying drug delivery systems (S-SNEDDS) partially bypass these gastrointestinal uncertainties by improving absorption (34, 35), but do not resolve the underlying question of how unformulated or endogenously occurring MacDGs are processed in the gut. Resolving these digestive, absorptive, and microbial fate questions is a prerequisite for understanding the pharmacokinetic behavior and systemic bioavailability of MacDGs in therapeutic contexts.

## 3 Natural occurrence and biological presence of monoacetyldiacylglycerols

Monoacetyldiacylglycerols have been detected across diverse biological contexts, including fermented foods, marine organisms, insects, and mammalian tissues (**Table 1**). Despite their broad distribution, their physiological significance and endogenous status in humans remain uncertain.

**Table 1:** Natural occurrence of monoacetyldiacylglycerols across biological systems.

Source	Examples	Concentration	Function	References
Fermented dairy products	Cheese, cultured milk	Trace levels	Microbial lipid modification during fermentation	(37)
Mammalian tissues	Deer antler, moose antler, bovine udder, moose/caribou muscle	Up to ~1–4% of total triglycerides	Growth- and regeneration-associated lipid pools (proposed)	(28, 30, 31)
Marine organisms	Fish, marine invertebrates	Trace to low abundance	Membrane remodelling under environmental stress (putative)	(38)
Insects	Goldenrod gall fly larvae	Moderate (% of total lipids)	Cryoprotection and membrane stabilization	(38)
Plant oils and seeds	Canola (boreal climates), Euonymus seeds	Variable	Stress adaptation and seed lipid metabolism (proposed)	(29, 39)

### 3.1 Occurrence in food systems

MacDGs occur in fermented dairy products at low concentrations (μg/g range) through microbial lipase, esterase, and acyltransferase activities (28, 37, 40). Given limited oral bioavailability (discussed in Section 2.4), significant systemic exposure from dietary sources appears unlikely without specialized formulations.

### 3.2 Presence in marine, insect, and mammalian tissues

MacDGs have been detected in marine organisms and insects, often associated with stress adaptation, membrane remodeling, and environmental challenges. The goldenrod gall fly (*Eurosta solidaginis*), for example, accumulates MacDGs during larval development prior to winter, where they may contribute to cryoprotective mechanisms (38).

MacDG detection in cervid tissues, including antler extracts, during phases of rapid growth and regenerative activity is particularly intriguing (30, 31). MacDG concentrations in moose antlers reach approximately 4% of total triglycerides. Critically, natural deer antler-derived MacDG extracts demonstrated hematopoietic stem cell-stimulating activity (41), yet such activity has not been demonstrated for synthetic PLAG. This divergence could reflect: (1) structural differences between complex natural MacDG mixtures and pure PLAG, (2) presence of bioactive co-constituents in natural extracts, (3) concentration-dependent effects not replicated in synthetic studies, or (4) tissue-specific metabolic activation requirements. Resolving this discrepancy through comparative structural analysis would illuminate whether synthetic MacDGs recapitulate or fundamentally diverge from natural MacDG biology.

### 3.3 Implications for endogenous biology and translational development

The detection of MacDGs across varied biological systems raises important questions regarding their status in humans. The appearance of MacDGs in mammalian tissues may reflect regulated biosynthetic events during specific physiological states (growth, regeneration, lactation), transient synthesis during metabolic stress, environmental exposure through diet, or incidental products of neutral lipid remodeling. This ambiguity has direct implications for translational development. If MacDGs are intentionally synthesized as part of an unrecognized lipid mediator pathway, their therapeutic use would represent augmentation of endogenous anti-inflammatory mechanisms. Conversely, if they arise primarily from diet or microbial metabolism, their therapeutic application represents pharmacological exploitation of bioactive xenobiotics.

## 4 Mechanisms of action of monoacetyldiacylglycerols

Monoacetyldiacylglycerols exert immunomodulatory and cytoprotective effects across epithelial, myeloid, and stromal compartments. Although the majority of mechanistic evidence derives from the synthetic analog PLAG, the reproducibility of several signatures across experimental systems supports the emergence of a coherent mechanistic profile.

### 4.1 Suppression of JAK–STAT signaling: inhibition of STAT3 and STAT6 activation

One of the most robust mechanistic signatures associated with MacDG activity is the attenuation of the JAK–STAT pathway, particularly the signaling cascades centered on STAT3 and STAT6 (**Figure 2**). Across epithelial cells, macrophages, T lymphocytes, and other cytokine-responsive cell types, these compounds reduce ligand-induced phosphorylation of STAT3 and STAT6, limit their nuclear translocation, and dampen the downstream transcriptional programs that propagate inflammatory responses.

**Figure 2:**
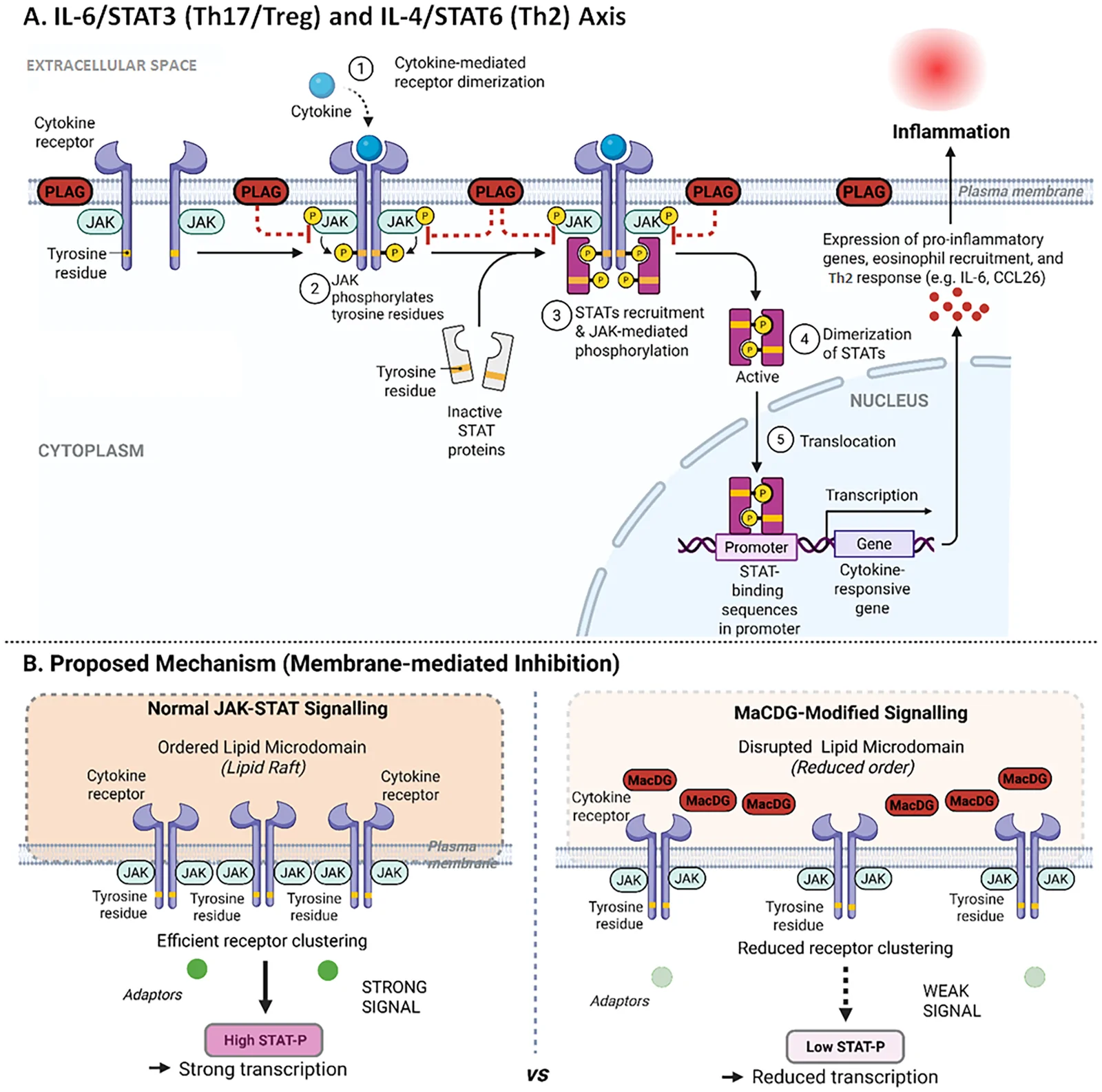
Proposed membrane-centric modulation of cytokine-driven JAK/STAT signaling by PLAG (MacDG analog), with T-helper axis relevance indicated. **(A)** Canonical IL-6/STAT3 and IL-4/STAT6 activation involves cytokine binding, receptor dimerization, JAK activation, and STAT phosphorylation, culminating in transcription of pro-inflammatory genes. The IL-6/STAT3 arm is most directly relevant to the Th17/Treg axis, through which persistent STAT3 signaling promotes Th17 differentiation and suppresses Foxp3^+^ Treg expansion; the IL-4/STAT6 arm is most directly relevant to the Th2 axis, driving eosinophilic and atopic inflammatory responses. PLAG is hypothesized to intercalate into the plasma membrane, where it may alter ordered lipid microdomains, reduce receptor clustering, and limit downstream STAT activation across both axes. **(B)** Working model comparing normal receptor organization with PLAG-modified conditions, in which membrane remodeling may attenuate inflammatory signaling. This schematic represents a proposed mechanism with potential implications for inflammatory disease. Created in BioRender. Tao, L. (2026) https://BioRender.com/hr5nryu.

#### 4.1.1 STAT3 in chronic inflammatory pathology

Among STAT family members, STAT3 plays a central role in chronic inflammatory pathology, responding to IL-6 and related cytokines to promote Th17 polarization, fibroblast activation, and sustained tissue injury (42, 43). Persistent STAT3 signaling contributes to refractory inflammation in rheumatoid arthritis, colitis, and several epithelial inflammatory disorders.

Reduction in STAT3 phosphorylation following MacDG treatment has been consistently observed *in vitro* and *in vivo*, corresponding with diminished expression of downstream inflammatory mediators including IL-6 and attenuated tissue damage in models of lung injury, colitis, and autoimmune arthritis (16, 18, 44).

#### 4.1.2 STAT6 in allergic and eosinophilic inflammation

STAT6, activated primarily by IL-4 and IL-13, plays a key role in eosinophilic inflammation, mucus hypersecretion, fibrotic progression, and airway remodeling (45). It controls expression of genes implicated in eosinophil recruitment through upregulation of pro-inflammatory chemokines such as CCL26. The inhibition of STAT6 disrupts transcriptional programs underlying these pathological processes and reduces eosinophilic infiltration, complementing STAT3-dependent modulation of chronic inflammation (46, 47).

Yoon et al. demonstrated that MacDG attenuated IL-4 expression and modulated Th2 immunity in allergen-stimulated cell and murine models, underscoring a direct role for these compounds in controlling IL-4–dependent allergic inflammation via STAT6 modulation. The parallel attenuation of STAT3 and STAT6 positions MacDGs within a rare category of lipid-derived molecules capable of influencing multiple STAT-dependent pathways simultaneously (48).

#### 4.1.3 Rebalancing adaptive immunity: Th17/Treg dynamics through STAT3 inhibition

MacDG-mediated STAT3 suppression extends beyond innate immunity into adaptive immune regulation. Of particular significance is the reciprocal relationship between pro-inflammatory Th17 cells and anti-inflammatory regulatory T cells (Tregs). This Th17/Treg equilibrium is essential for preventing autoimmunity and chronic inflammatory pathology (49, 50). Th17 differentiation, driven by IL-6/STAT3 signaling, results in IL-17A secretion and tissue destructive inflammation, whereas Foxp3^+^ Tregs restrain excessive immune activation and maintain peripheral tolerance (51).

Preclinical evidence indicates that MacDGs directly modulate CD4^+^ T cell fate through STAT3 inhibition. In murine splenic CD4^+^ T cells cultured under Th17-polarizing conditions, PLAG markedly reduced IL-17A secretion, downregulated RORC expression, and decreased STAT3 (Tyr705) phosphorylation without compromising cell viability (18). Under Treg-polarizing conditions, PLAG enhanced Foxp3^+^ expression and increased CD25^+^ Foxp3^+^ cells. These findings extend to human peripheral blood mononuclear cells from both healthy donors and rheumatoid arthritis patients. PLAG treatment ex vivo decreased IL-17 and IL-6 secretion, reduced IL-17^+^ CD4^+^ cells, and increased CD25^+^ Foxp3^+^ Tregs (18). Correspondingly, RORC mRNA levels declined, Foxp3 mRNA increased, and phosphorylated STAT3 protein levels were diminished. These human validation studies underscore translational relevance of PLAG-mediated Treg/Th17 rebalancing and suggest therapeutic potential in autoimmune contexts.

Supporting evidence from *in vivo* models demonstrates that PLAG administration suppresses IL-6/STAT3 signaling, limits neutrophil recruitment, and reshapes cytokine networks in murine models of collagen-induced arthritis, autoimmune lung inflammation, and atopic dermatitis, collectively fostering a Treg-favoring immune milieu (18, 44, 52). However, lineage-tracing, adoptive-transfer, and selective Treg-depletion studies are needed to confirm whether Treg/Th17 reprogramming represents a T-cell autonomous mechanism or a secondary consequence of altered cytokine environments *in vivo* (53, 54). Overall, these compounds appear to establish a tolerogenic immune environment through coordinated regulation of innate and adaptive immune pathways, with STAT3 inhibition serving as a unifying molecular mechanism across multiple immune cell lineages.

Collectively, these preclinical and translational studies demonstrate that MacDGs exert broad antiinflammatory and immunoregulatory effects by inhibiting STAT3- and STAT6-mediated pathways across innate and adaptive immune compartments. STAT3 suppression limits IL-6–driven neutrophilic inflammation and rebalances Th17/Treg dynamics, while STAT6 inhibition attenuates IL-4–dependent eosinophilic and Th2 responses. Through this dual action on multiple STAT pathways, these compounds modulate Th2- and Th17-driven inflammation and adaptive immune tolerance, providing a mechanistic basis for their therapeutic potential in chronic inflammatory, allergic, and autoimmune diseases.

### 4.2 Modulation of NF-κB signaling and downstream cytokine networks

Nuclear factor kappa-light-chain-enhancer of activated B cells (NF-κB) is a master regulator of inflammatory gene expression (55, 56). NF-κB activation, triggered by pattern recognition receptors such as Toll-like receptor 4 (TLR4) upon recognition of bacterial lipopolysaccharide, requires phosphorylation and degradation of inhibitor of κBα (IκBα), allowing NF-κB subunits (p65/p50) to translocate into the nucleus to initiate transcription of genes involved in inflammation and immune responses (**Figure 3**).

**Figure 3:**
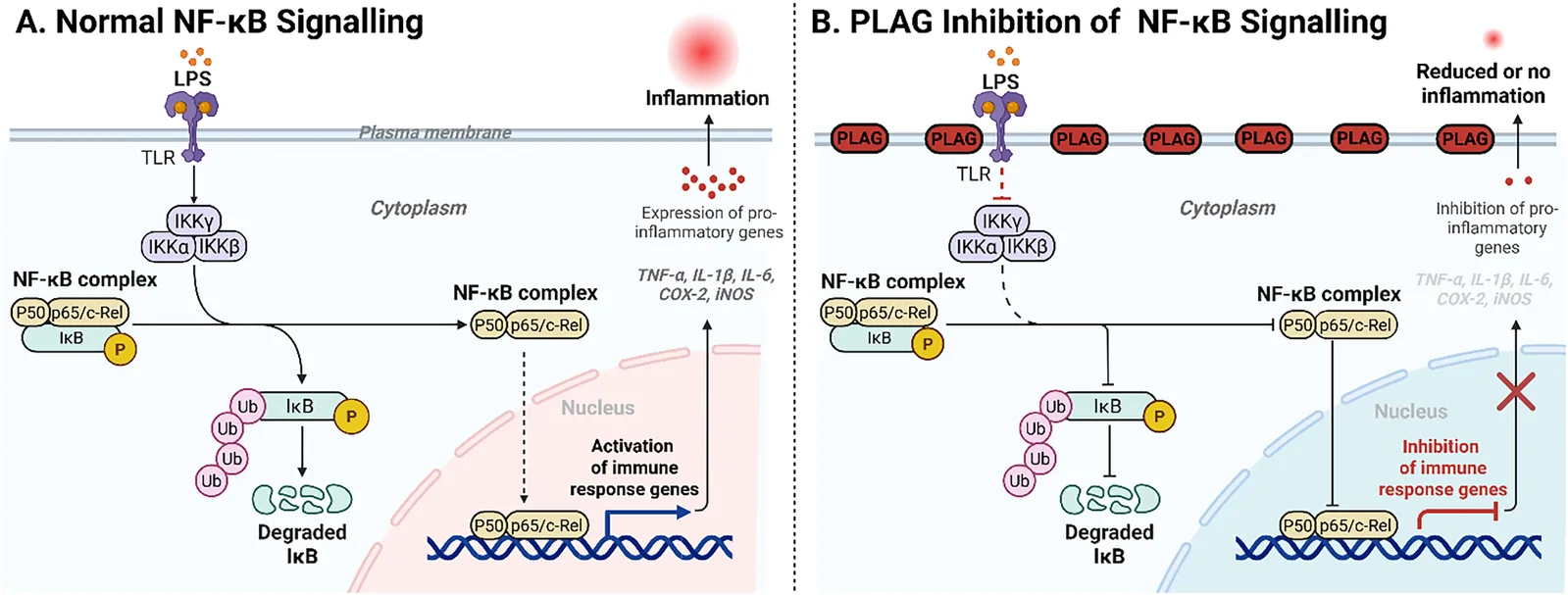
Proposed membrane-centric modulation of NF-κB signaling by PLAG (MacDG analog). **(A)** Canonical NF-κB activation is initiated by LPS–TLR4 engagement, leading to IKK activation, phosphorylation and degradation of IκB, and nuclear translocation of NF-κB (p50/p65/c-Rel) to promote transcription of pro-inflammatory genes. **(B)** PLAG is hypothesized to intercalate into the plasma membrane and alter receptor-associated signaling, potentially limiting NF-κB nuclear activity and attenuating inflammatory gene expression. This schematic represents a proposed mechanism with potential implications for inflammatory disease. Created in BioRender. Tao, L. (2026) https://BioRender.com/3dqppgg.

Across multiple inflammatory models, PLAG suppresses NF-κB activation by reducing IκB degradation and preventing p65 nuclear translocation (17, 57). In lipopolysaccharide-challenged macrophages and tumor necrosis factor-alpha (TNF-α)-stimulated epithelial cells, PLAG attenuates NF-κB-dependent cytokine production, including IL-6 and IL-1β (16, 17). MacDG-induced reductions in IKK activation, stabilization of IκBα, and diminished nuclear translocation of p65/p50 complexes collectively contribute to decreased transcription of proinflammatory cytokines. The significance of NF-κB modulation extends beyond cytokine suppression. In epithelial cells, attenuation of NF-κB activity reduces apoptosis, limits oxidative stress-induced injury, and preserves barrier integrity during inflammatory challenge (16). In macrophages, reduced NF-κB signaling facilitates repolarization away from classical M1 phenotypes and supports resolution-associated programs (17). The breadth of NF-κB-related outcomes underscores the multinode nature of MacDG action.

The mechanism underlying MacDG-mediated NF-κB suppression remains incompletely defined but appears distinct from classical IκB kinase inhibitors. Rather than blocking upstream kinase cascades, MacDGs may interfere with receptor-proximal signaling platforms or alter membrane microdomain organization that facilitates NF-κB activation. Importantly, the timing and magnitude of NF-κB inhibition do not appear downstream of STAT attenuation but rather operate as a parallel mechanistic axis. This pattern suggests that MacDGs influence multiple membrane-proximal signaling complexes that govern innate immune activation. The parallel suppression of NF-κB and JAK-STAT pathways positions these compounds as dual-axis immunomodulators capable of attenuating inflammatory responses initiated by diverse upstream triggers.

### 4.3 Membrane-proximal modulation and microdomain-linked interference

Multiple MacDG effects across inflammatory pathways are difficult to attribute to single ligand–receptor interactions, suggesting an upstream membrane-associated mechanism that modulates receptor organization and trafficking. This pattern of pleiotropic activity, affecting diverse receptors across multiple cell types, points toward modulation of a shared proximal context that shapes receptor coupling to intracellular adaptors rather than direct engagement of individual binding sites.

Several lines of evidence suggest that MacDGs exert immunomodulatory effects through membrane-associated mechanisms rather than direct receptor binding. First, PLAG has been shown to modulate P2Y2 receptor trafficking and stability in cancer cells, reducing inflammatory signaling without classical antagonism (58). Second, PLAG induces glucose transporter 2 (GLUT2) endocytosis in pancreatic β cells during streptozotocin-induced metabolic stress (59). Additionally, MacDGs influence adenosine A2B receptor degradation through thioredoxin-interacting protein (TXNIP)-dependent pathways (60). These findings demonstrate capacity to modulate receptor abundance and redistribution without direct receptor-ligand competition.

Natural MacDG occurrence in stress-adapted organisms and regenerating tissues (28, 30) supports a role in membrane remodeling under physiological stress. The distinctive sn-3 acetyl group disrupts classical triglyceride packing, potentially altering lipid microdomain organization. Unlike diacylglycerols, which activate PKC through C1 domain binding (25), MacDGs do not engage these classical lipid-sensing domains (27), suggesting alternative membrane-linked mechanisms (61, 62).

This microdomain-linked hypothesis posits that MacDG incorporation into cellular membranes alters the local lipid environment, influencing receptor partitioning, clustering, and access to signaling machinery. Lipid rafts and microdomains are established determinants of receptor signaling efficiency and therapeutically relevant platforms (63, 64). Classical membrane studies demonstrate that lipid structural features modify membrane properties and protein function (25). However, direct evidence for MacDG-induced remodeling of rafts or microdomains, including partitioning, diffusion dynamics, or curvature effects, remains unestablished and represents hypothesis-generating territory requiring biophysical validation through super-resolution imaging, fluorescence recovery techniques, and molecular dynamics simulations.

### 4.4 Modulation of macrophage functional states toward resolution-associated programs

A significant body of evidence indicates that MacDGs modulate macrophage behavior in a manner consistent with inflammation resolution, without enforcing a fixed transition toward canonical M2 phenotypes. Across *in vitro* and *in vivo* systems, treatment with these compounds reproducibly suppresses macrophage-derived inflammatory outputs linked to oxidative stress and chemokine-driven leukocyte recruitment, while promoting functions associated with tissue protection and inflammatory clearance.

In macrophage-lineage cells exposed to inflammatory or chemotherapeutic stressors, PLAG attenuates NADPH oxidase 2 (NOX2)–dependent reactive oxygen species (ROS) generation and downstream mitogen-activated protein kinase (MAPK) signaling, resulting in reduced transcription and secretion of neutrophil-attracting chemokines, including CXCL8 and its murine homologue MIP-2 (65, 66). These effects arise from inhibition of upstream PLCβ3–PKC signaling and impaired membrane translocation of NOX2 cytosolic subunits such as Rac1 and phosphorylated p47phox.

These effects translate *in vivo* into coordinated immunomodulation, including preserved circulating neutrophil counts and limited tissue neutrophil accumulation (67). Despite these consistent functional effects, systematic assessment of canonical M2 markers, including CD206, Ym1, Fizz1, or Arg1, has not yet been performed, precluding definitive assignment of a fully polarized macrophage phenotype (68, 69).

#### 4.4.1 Enhancement of efferocytic clearance and pro-resolving activity

Beyond phenotypic markers, functional capacity for resolution can be assessed through effector mechanisms. A mechanistically distinct feature is the enhancement of efferocytosis—the phagocytic clearance of apoptotic cells that is essential for inflammation resolution. PLAG promotes efferocytic clearance of apoptotic neutrophils through membrane microdomain remodeling and P2Y2 receptor redistribution, coupled with cytoskeletal reorganization and increased interleukin-10 (IL-10) secretion (58).

As effective efferocytosis reinforces anti-inflammatory signaling and prevents secondary necrosis that would amplify tissue injury, this represents a pro-resolving mechanism distinct from simple inflammatory suppression (70). The enhancement of efferocytosis places MacDG activity in the same functional category as specialized pro-resolving mediators (SPMs), in that both actively promote inflammation resolution rather than merely blocking inflammatory initiation (71, 72); however, this parallel is descriptive rather than mechanistic. No COX-2- or ALOX-12/15-dependent biosynthetic or signaling link has been established for MacDGs, and whether their pro-resolving functional profile reflects convergence on shared downstream programs or an entirely independent upstream mechanism remains an open question for future investigation.

Collectively, MacDG-mediated macrophage reprogramming appears to arise from convergent modulation of intracellular signaling, membrane organization, and potentially metabolic or lipid-handling pathways, although these latter dimensions remain incompletely defined (17, 58, 73, 74). At the tissue level, these macrophage-centered effects intersect with reduced neutrophil infiltration, preservation of epithelial barrier integrity, and attenuation of oxidative and proteolytic injury across multiple inflammatory disease models (16, 17, 75, 76). Together, these data support a model in which these compounds act as multinodal regulators of innate immunity, promoting resolution-oriented macrophage behavior while highlighting key mechanistic gaps that warrant further investigation.

### 4.5 Context-dependent modulation of neutrophil trafficking and resolution

An emerging integrative feature of MacDG biology is the context-dependent modulation of neutrophil dynamics, in which excessive neutrophil recruitment and activation are restrained during acute inflammatory responses while physiological neutrophil availability is preserved under inflammatory or chemotherapeutic stress (77). Rather than acting as direct inhibitors or stimulators of neutrophil function, these lipid modulators appear to influence neutrophil behavior indirectly by reshaping chemokine gradients, oxidative signaling, and tissue–leukocyte interactions within inflamed microenvironments.

During acute inflammation, MacDG administration consistently limits pathological neutrophil accumulation at sites of tissue injury (16, 78, 79). Across experimental models of acute lung injury and gout-like inflammation, PLAG accelerates clearance of neutrophil-attracting chemokines (17, 80). These effects are accompanied by reduced neutrophil accumulation, diminished ROS production, and decreased NET formation.

Mechanistically, restraint of neutrophil recruitment reflects coordinated modulation of upstream inflammatory signaling rather than direct interference with neutrophil receptors. In myeloid and epithelial compartments, PLAG attenuates NF-κB–dependent chemokine production and limits NOX2-mediated oxidative amplification by inhibiting Rac1 membrane translocation and phosphorylation of p47phox (65, 66, 81). In parallel, accelerated receptor recycling and reduced persistence of inflammatory ligands normalize chemokine landscapes, lowering neutrophil recruitment pressure without abolishing basal immune surveillance.

Under chemotherapy-associated inflammatory stress, these compounds reduce inappropriate neutrophil extravasation and oxidative activation, preserving circulating neutrophil pools. In 5fluorouracil/gemcitabine-treated murine models, PLAG limits tissue sequestration of neutrophils, resulting in maintained peripheral neutrophil counts without inducing excessive inflammatory rebound (65, 82, 83). These findings indicate that normalization of chemokine and redox signaling supports physiological neutrophil availability, without evidence for direct stimulation of neutrophil production. Direct effects of MacDGs on granulopoiesis or bone-marrow neutrophil output have not been demonstrated.

#### 4.5.1 Clinical evidence: chemotherapy-induced neutropenia

Pilot clinical evidence from pancreatic cancer patients undergoing gemcitabine-based chemotherapy demonstrated that oral PLAG administration was associated with reduced incidence of chemotherapy-induced neutropenia compared to historical controls (21), suggesting clinical translation of neutrophil-preserving effects observed in preclinical models. These preliminary findings support further investigation of MacDGs as potential adjunctive agents in chemotherapy-related myelosuppression.

#### 4.5.2 Tumor microenvironment normalization and immune checkpoint synergy

In tumor models, MacDG-mediated reduction of tumor-associated neutrophils (TANs) demonstrates additional therapeutic relevance beyond acute inflammation. TANs can suppress anti-tumor immunity through multiple mechanisms including generation of immunosuppressive mediators, inhibition of T-cell infiltration, and promotion of angiogenesis. PLAG treatment reduces TAN infiltration in murine models of non-small cell lung carcinoma and bladder cancer, correlating with improved tumor control (60, 78, 79).

Critically, this neutrophil normalization enhances efficacy of immune checkpoint blockade. In syngeneic tumor models, combination of PLAG with anti-programmed cell death protein 1 (antiPD-1) or anti-programmed death-ligand 1 (anti-PD-1) antibodies resulted in synergistic antitumor effects superior to checkpoint inhibitors alone (79, 84). The mechanism appears to involve reduction of immunosuppressive myeloid populations that limit T-cell infiltration and tumor recognition. By normalizing the myeloid compartment, these compounds unmask tumors to checkpoint blockade therapy, positioning them as potential adjuvants in cancer immunotherapy operating through immune normalization rather than direct cytotoxicity (78).

Together, these observations support a model in which MacDGs regulate neutrophil dynamics in a context-dependent manner by indirectly tuning inflammatory cues. By restraining excessive neutrophil infiltration during acute inflammation while preserving neutrophil availability under chemotherapeutic stress and normalizing tumor-associated myeloid populations, these lipid modulators promote resolution-oriented immune responses and limit collateral tissue damage. This regulatory profile provides a mechanistic rationale for evaluating MacDGs in diseases characterized by neutrophil-driven pathology, including acute lung injury, gout, colitis, chemotherapy-associated neutropenia, and as adjuvants to cancer immunotherapy.

### 4.6 Epithelial barrier stabilization

An important and increasingly plausible component of MacDG activity is functional protection of epithelial-dominated tissues during inflammatory and oxidative stress. Across airway and mucosal injury models, these compounds reduce inflammatory burden and tissue damage in ways that preserve tissue-level function without necessarily acting directly on epithelial tight junctions or barrier structures (17, 83, 85).

The most direct evidence for epithelial involvement is PLAG’s suppression of CCL26 expression in epithelial cells, which constrains eosinophil chemotaxis and demonstrates that MacDGs can directly modulate epithelial inflammatory signaling (46). However, whether this extends to broader cytoprotective effects or barrier stabilization remains unclear. Protective effects in mucosal injury settings such as oral mucositis further support a role for these compounds in limiting epithelial tissue injury under cytotoxic stress, although precise junctional determinants have not been uniformly characterized (19, 83).

#### 4.6.1 Clinical evidence: oral mucositis

In a Phase 2, multicenter, randomized, double-blind, placebo-controlled trial, EC-18 demonstrated significant efficacy in reducing severity and duration of oral mucositis secondary to chemoradiation therapy in head and neck cancer patients (19). This represents the most robust clinical validation of MacDG epithelial protective effects to date, providing human evidence that the tissue-protective mechanisms observed in preclinical models translate to clinical benefit.

Mechanistically, epithelial protection can be framed as an integrated consequence of immune–tissue crosstalk rather than established tight-junction stabilization per se. Experimental findings support a hierarchical model. First, attenuation of NF-κB- and STAT-linked inflammatory signaling reduces epithelial and stromal cytokine production, lowering paracrine amplification of injury (42, 55, 86), the same anti-inflammatory mechanism operating in immune cells, now demonstrated in epithelial populations. Second, by limiting neutrophil (and eosinophil) recruitment through reduced chemokine gradients, these lipid modulators decrease epithelial exposure to proteases, ROS, and extracellular traps that disrupt epithelial architecture and delay repair (17, 77, 80). Third, epithelial-intrinsic cytoprotective effects remain plausible, particularly under oxidative stress, but definitive evidence for mitochondrial preservation or tight-junction reinforcement is currently limited and will require dedicated barrier assays (87, 88).

Collectively, available data support a model in which these compounds protect epithelial-rich tissues primarily by dampening inflammatory signaling and reducing leukocyte-driven collateral injury, with potential additional epithelial-intrinsic contributions that remain to be resolved. The established direct effect, suppression of epithelial chemokine expression, demonstrates that epithelial cells are legitimate MacDG targets, but whether this translates to direct cytoprotection, mitochondrial preservation, or tight junction stabilization awaits dedicated investigation.

### 4.7 Modulation of osteoclastogenic signaling in inflammatory bone disease

MacDGs also appear to modulate bone-destructive inflammatory programs relevant to erosive arthritis. In autoimmune arthritis models, EC-18/PLAG administration attenuates joint inflammation and has been reported to suppress osteoclastogenic responses, aligning with reduced structural damage in settings where receptor activator of nuclear factor kappa-B ligand (RANKL)-driven osteoclast differentiation is central to pathology (44, 89, 90). This bone-protective profile is mechanistically consistent with the broader MacDG-associated dampening of NF-κB and STAT3 signaling described above (Sections 4.1–4.2), pathways that cooperate downstream of inflammatory cytokines and RANKL to promote osteoclast maturation and the expression of bone-resorptive enzymes.

However, the level at which these compounds act within the osteoclastogenic axis remains unresolved, as current evidence does not distinguish direct effects on osteoclast precursors from secondary consequences of altered cytokine networks and stromal–myeloid crosstalk within inflamed synovium. Clarifying receptor-proximal signaling events and lineage-resolved responses will be necessary to determine whether osteoclast suppression represents a primary MacDG mechanism or a downstream correlate of upstream immunomodulation.

### 4.8 Emerging and hypothesis-generating mechanisms

Several mechanistic pathways have been proposed on the basis of preliminary findings, although none have yet achieved the consistency required for firm mechanistic attribution. Among these is the potential engagement of the nuclear factor erythroid 2-related factor 2 (Nrf2) cytoprotective pathway, suggested by increased expression of antioxidant genes and enhanced resistance to oxidative injury in select epithelial models. Activation of Nrf2 may reflect a secondary consequence of reduced oxidative and inflammatory stress or a more direct interaction with redox-sensitive signaling nodes (87, 88).

There is also limited evidence suggesting that MacDGs may penetrate the central nervous system under certain conditions, although the functional relevance of this observation is unknown. If confirmed, such penetration could implicate roles in neuroinflammation or blood–brain barrier biology, but current data are too sparse to support definitive conclusions.

Early studies identified monoacetyldiglycerides purified from deer antler as hematopoietic stem cell-stimulating agents (31, 41). Whether synthetic MacDGs such as PLAG retain this activity, and how it relates to the neutrophil-preserving effects observed under chemotherapeutic stress, remains unexplored. If validated, direct hematopoietic stimulation would represent a mechanism distinct from the indirect neutrophil preservation attributed to reduced tissue sequestration and inflammatory stress. However, no direct evidence for granulopoietic stimulation by synthetic MacDGs has been demonstrated in bone marrow studies.

### 4.9 Conceptual integration: a multi-node modulator of inflammation

Taken together, the mechanistic profile of MacDGs suggests a lipid mediator capable of acting at multiple hierarchical levels of the inflammatory response (**Table 2**). By simultaneously modulating the JAK–STAT and NF-κB pathways, recalibrating neutrophil and macrophage behaviors, rebalancing Th17/Treg adaptive immunity, and preserving epithelial integrity, these compounds achieve a coordinated anti-inflammatory effect that is broader than would be expected from the inhibition of a single cytokine or receptor. This multinode regulatory capacity may account for their efficacy across various preclinical models, ranging from autoimmune inflammation to acute lung injury and chemotherapy-induced myelosuppression.

**Table 2:** Summary of MacDG mechanisms of action and supporting evidence.

Mechanism	Primary pathway(s)	Key mediators/outcomes	Disease models validated	Evidence quality	Human validation	Mechanism certainty	Th-axis relevance	Key references
JAK-STAT suppression	STAT3, STAT6	IL-6↓, IL-4↓, CCL26↓	Arthritis, asthma, colitis, AD	High	Yes (cellular assays)	Established	Th17/Treg (STAT3); Th2 (STAT6)	(16, 17, 46, 52)
Th17/Treg rebalancing	STAT3 in CD4^+^ T cells	IL-17A↓, RORC↓, Foxp3↑	Arthritis, lung inflammation, AD	High	Yes (RA patients, ex vivo)	Established	Th17/Treg (direct)	(18)
NF-κB inhibition	IκB/p65 translocation	IL-1β↓, IL-6↓	ALI, mucositis, sepsis, arthritis	High	Indirect (mucositis trial)	Established	Th1-associated (partial; IL-6 and IL-1β↓ documented without TNF-α suppression); M1 repolarization documented; IFN-γ and T-bet effects unestablished; innate predominant	(17, 83)
Membrane-proximal modulation	Receptor trafficking	P2Y2, GLUT2, A2B receptors	Varied contexts	Moderate	No	Hypothetical	Not Th-axis specific; upstream of lineage specification	(60)
Neutrophil regulation	Chemokine gradients, NOX2	CXCL8/MIP-2↓, ROS↓, NETs↓	ALI, gout, CIN, tumours	High	Yes (CIN pilot study)	Probable	Th17-associated (indirect: CXCL8/IL-17 axis crosstalk); innate-predominant	(17)
Macrophage reprogramming	NOX2, efferocytosis, P2Y2	ROS↓, IL-10↑, apoptotic clearance	Multiple inflammatory models	Moderate	No	Probable	Th17 (indirect via IL-6↓); Th2-associated (IL-10↑; canonical M2 markers not assessed); innate-predominant	(58)
Epithelial protection	Indirect (immune dampening)	CCL26↓, tissue damage↓	Mucositis, ALI, asthma	High	Yes (Phase 2 mucositis)	Probable (indirect)	Th2-relevant in atopic/allergic contexts (CCL26/IL-4/IL-13 axis); innate-predominant	(46, 47, 83)
Osteoclast suppression	NF-κB/STAT3 (presumed)	RANKL-driven differentiation	Autoimmune arthritis	Moderate	No	Unclear (indirect)	Th17-associated (indirect; RANKL/IL-17 osteoclastogenesis axis)	(44)

Arrows indicate direction of effect (↓, decreased, ↑, increased). Abbreviations: AD, atopic dermatitis; ALI, acute lung injury; CIN, chemotherapy-induced neutropenia; IL, interleukin; NETs, neutrophil extracellular traps; NF-κB, nuclear factor kappa-light-chain-enhancer of activated B cells; NOX2, NADPH oxidase 2; RA, rheumatoid arthritis; RANKL, receptor activator of nuclear factor kappa-B ligand; ROS, reactive oxygen species; RORC, RAR-related orphan receptor C; STAT, signal transducer and activator of transcription; TNF-α, tumor necrosis factor-α.

Considered against classical T-helper immune axes, the mechanisms described above reveal a differentiated pattern of adaptive and innate immune engagement. MacDG-mediated STAT3 suppression most directly implicates the Th17/Treg axis; attenuation of IL-6/STAT3-driven Th17 differentiation, reduced IL-17A secretion, and enhanced Foxp3^+^ Treg expansion collectively position these compounds as modulators of Th17-predominant autoimmune and chronic inflammatory pathology (Sections 4.1.1, 4.1.3) (91, 92). STAT6 inhibition most directly implicates the Th2 axis; attenuation of IL-4- and IL-13-driven responses, including CCL26 suppression and eosinophil chemotaxis reduction, is consistent with modulation of Th2-polarized allergic and atopic disease (Section 4.1.2) (93, 94). MacDG effects on Th1-associated responses are partially supported but require qualification; while NF-κB suppression and macrophage repolarization away from M1 phenotypes are documented (Sections 4.2, 4.4), selective suppression of IL-6 and IL-1β without affecting TNF-α (17) suggests these effects are more accurately characterized as partial dampening of M1-associated outputs than as broad Th1 suppression (95, 96). Direct evidence for MacDG modulation of IFN-γ production, T-bet expression, or classical Th1 T-cell polarization has not been established and remains a priority for future investigation. Neutrophil regulatory effects, while innate in origin, are not entirely Th-axis independent; CXCL8 suppression intersects with the well-characterized Th17-neutrophil crosstalk axis, through which Th17-derived IL-17A induces CXCL8 expression and neutrophil recruitment, and neutrophils reciprocally chemoattract Th17 cells via CCL20 (Section 4.5) (97, 98). Mechanisms operating through membrane microdomain modulation and epithelial barrier stabilization function substantially upstream of and independently from T-helper lineage specification; their effects on Th-axis balance are indirect, mediated through downstream cytokine milieu changes rather than direct T-helper polarization (Sections 4.3, 4.6) (99).

However, the absence of a known primary molecular target remains a central challenge. Whether these compounds act through a specific receptor, membrane–lipid microenvironment alteration, or yet-unidentified metabolic sensor is unknown. The convergent evidence points toward membrane-proximal mechanisms that reshape receptor microenvironments and modulate multiple signaling cascades simultaneously, positioning MacDGs as noncanonical lipid modulators distinct from classical diacylglycerol signaling molecules. Resolving this foundational question will be essential for validating the mechanisms described above, optimizing structure–activity relationships, predicting off-target effects, and establishing MacDGs as a mechanistically coherent therapeutic class.

## 5 Therapeutic applications across disease domains

The mechanistic breadth of monoacetyldiacylglycerols, exemplified predominantly by PLAG/EC-18, has prompted evaluation across inflammatory and injury-associated disease models. Although the therapeutic landscape remains largely preclinical, with notable exceptions including ongoing Phase 2 trials in atopic dermatitis (NCT06487000) and preliminary clinical data in chemotherapy-associated toxicities, the reproducibility of MacDG effects provides a coherent rationale for exploring their translational relevance. Disease domains are ordered according to mechanistic coherence, reproducibility across models, and translational maturity (**Table 3**).

**Table 3:** Therapeutic applications of MacDGs across disease domains: evidence summary and translational priorities.

Disease domain	Mechanistic alignment	Evidence level	Clinical status	Key mechanisms	Translational priority
Acute lung injury	High	Replicated preclinical models	None	STAT3/NF-κB suppression; normalization of neutrophil trafficking; epithelial protection	High
Asthma/allergic airway disease	High	Multiple independent preclinical models	None	STAT6 inhibition; CCL26 suppression; eosinophil regulation	High
Atopic dermatitis	High	Preclinical + Phase 2 ongoing	NCT06487000 (active)	JAK1/STAT6 suppression; Th2 cytokine attenuation; barrier support	Highest
Rheumatoid arthritis	Moderate	Preclinical autoimmune arthritis models	None	IL-6/STAT3 and NF-κB suppression; indirect modulation of osteoclastogenesis	Moderate
Mucositis/neutropenia	High	Preclinical + pilot clinical data	Early RCTs completed	Normalization of neutrophil trafficking; epithelial repair; stromal signalling modulation	Highest
Immune-mediated hepatic inflammation	Moderate	Single experimental model (ConA hepatitis)	None	IL-4/STAT6 suppression; CXCL2-mediated neutrophil control	Moderate
Tumour-associated inflammation	Moderate	Multiple tumour models	None	P2Y2/TXNIP–A2BR-dependent myeloid normalization; enhancement of checkpoint inhibitor efficacy	Moderate
Neuroinflammation-associated cognitive dysfunction	Low (exploratory)	Limited; indirect evidence only	None	Acetylcholinesterase inhibition; LTP enhancement (inflammatory relevance unclear)	Low

ConA, concanavalin A; CXCL2, C-X-C motif chemokine ligand 2; JAK1, Janus kinase 1; LTP, long-term potentiation; NF-κB, nuclear factor kappa-light-chain-enhancer of activated B cells; RCT, randomized controlled trial; STAT, signal transducer and activator of transcription; TXNIP, thioredoxin-interacting protein; A2BR, adenosine A2B receptor.

### 5.1 Acute lung injury and inflammatory lung disease

Acute lung injury (ALI) and related inflammatory lung disorders are characterized by excessive neutrophil recruitment, epithelial and endothelial barrier disruption, cytokine amplification, and oxidative stress. Across multiple murine models, including lipopolysaccharide-induced lung injury and cigarette smoke–associated inflammation, PLAG administration consistently reduces pulmonary edema, neutrophil infiltration, and inflammatory cytokine production, accompanied by suppression of STAT3 and NF-κB signaling in airway epithelial cells and macrophages (17, 85). In autoimmune arthritis models with secondary lung involvement, PLAG also attenuates interstitial lung inflammation, suggesting relevance to autoimmune-associated inflammatory lung disease (18). Collectively, lung inflammation represents one of the most mechanistically coherent and experimentally replicated indications for MacDG intervention.

### 5.2 Asthma and allergic airway inflammation

Asthma and allergic airway disease are driven by Th2-skewed cytokine signaling, eosinophil recruitment, mucus hypersecretion, and epithelial stress. In allergen-induced murine asthma models, PLAG significantly reduces airway hyperresponsiveness, eosinophil infiltration, and Th2 cytokine expression, corresponding with attenuation of IL-4/STAT6 signaling in airway epithelial cells and macrophages (47, 48). Suppression of STAT6-dependent transcription and epithelial chemokine output, particularly CCL26, provides a direct link between MacDG exposure and reduced eosinophilic inflammation (46). These findings position allergic airway disease as a mechanistically well-supported indication, although long-term remodeling outcomes and human efficacy data remain limited.

### 5.3 Atopic dermatitis and Th2-mediated skin inflammation

Atopic dermatitis (AD) represents a Th2-dominant inflammatory skin disorder characterized by IL-4/IL-13 signaling, eosinophil infiltration, barrier dysfunction, and chronic pruritus. In chemically induced dermatitis models, oral PLAG administration reduces plasma IL-4 and immunoglobulin E (IgE) levels, diminishes eosinophil infiltration, and improves clinical and histological disease indices (52). These effects are accompanied by suppression of Janus kinase 1 (JAK1)/STAT6 phosphorylation within skin lesions, providing mechanistic coherence with the broader MacDG anti-inflammatory profile.

Importantly, a Phase 2 randomized, double-blind, placebo-controlled clinical trial evaluating oral PLAG in moderate-to-severe AD is currently underway (NCT06487000), making AD one of the most advanced non-oncologic translational indications for MacDGs. The trial design, patient selection criteria, and primary endpoints will provide critical evidence regarding MacDG efficacy in human Th2-mediated inflammation and inform dose optimization for future studies. Together, preclinical and emerging clinical data support Th2-mediated skin inflammation as a core disease domain for MacDG development (48, 52).

### 5.4 Rheumatoid arthritis and bone-destructive inflammation

Rheumatoid arthritis (RA) is characterized by persistent synovial inflammation, macrophage activation, cytokine amplification, and osteoclast-driven bone erosion. In murine models of autoimmune arthritis, PLAG reduces synovial inflammation, neutrophil infiltration, and joint destruction (16, 18, 44). These effects correlate with suppression of IL-6/STAT3 and NF-κB signaling, pathways that cooperate downstream of inflammatory cytokines and receptor activator of nuclear factor kappa-B ligand (RANKL) to promote osteoclastogenesis.

Experimental studies further report reduced osteoclast differentiation and preservation of trabecular bone architecture (44). However, whether osteoclast suppression reflects direct action on osteoclast precursors or secondary consequences of altered cytokine networks remains unresolved. Accordingly, bone protection is best viewed as a downstream correlate of upstream immunomodulation, rather than a primary MacDG mechanism.

### 5.5 Mucosal injury and chemotherapy-induced inflammation

Among all investigated indications, chemotherapy- and radiation-induced mucosal injury and neutropenia represent the most clinically advanced applications of MacDGs. In rodent models of oral mucositis, PLAG accelerates epithelial healing, reduces ulceration, and suppresses proinflammatory cytokines (83). Complementary murine studies demonstrate improved survival and preservation of intestinal architecture following total-body irradiation (100).

Clinically, early randomized and pilot studies in patients receiving gemcitabine or chemoradiation report reduced severity of neutropenia and mucositis, with favorable safety profiles (19, 21, 101). In a Phase 2 trial of oral mucositis in patients receiving high-dose chemotherapy, PLAG administration significantly reduced the duration and severity of mucositis compared to placebo, accelerated healing time, and improved quality of life measures (19). Mechanistic analyses from these studies indicate that clinical benefits correlate with reduced inflammatory cytokine levels and normalized neutrophil trafficking patterns.

Importantly, these effects appear to arise from normalization of neutrophil trafficking and stromal signaling rather than direct stimulation of granulopoiesis, distinguishing MacDGs from colony-stimulating factors such as granulocyte colony-stimulating factor (G-CSF). This mechanistic distinction has important implications for therapeutic positioning, particularly in oncology settings where excessive neutrophil stimulation may be undesirable. The combination of robust preclinical efficacy, mechanistic coherence, and positive early-phase clinical outcomes positions chemotherapy-associated mucosal injury as the most mature translational indication for MacDG therapeutics.

### 5.6 Immune-mediated hepatic inflammation

Immune-mediated hepatitis provides a context in which Th2 and neutrophil-driven inflammation converge on epithelial-rich tissues. In the concanavalin A–induced murine hepatitis model, PLAG confers marked hepatoprotection, reducing cytokine production, inflammatory cell infiltration, and tissue necrosis (102). These effects are linked to suppression of IL-4/STAT6 signaling and reduced C-X-C motif chemokine ligand 2 (CXCL2)-mediated neutrophil recruitment. These findings support MacDG activity in immune-mediated hepatic inflammation, though they should not be extrapolated to metabolic liver diseases in the absence of direct evidence.

### 5.7 Cancer-associated inflammation

Chronic inflammation contributes to tumor progression by fostering neutrophil-rich, immunosuppressive microenvironments. In multiple murine tumor models, including lung, bladder, and biliary cancers, PLAG reduces tumor-associated neutrophil accumulation, suppresses IL-6 and CXCL8 production, and enhances efficacy of immune checkpoint inhibitors (78, 79, 84, 103). These effects are linked to modulation of P2Y2 receptor trafficking and TXNIP-dependent degradation of adenosine A2B receptors, collectively reprogramming inflammatory tone within the tumor microenvironment (58–60). These data support MacDGs as adjunctive immunomodulators, not direct anticancer agents.

### 5.8 Neuroinflammation-associated cognitive impairment (exploratory)

Neurocognitive effects have been reported, but inflammatory endpoints were not directly measured in available studies, preliminary preclinical evidence in scopolamine-induced memory impairment models demonstrates that PLAG improves behavioral performance, reduces acetylcholinesterase activity, and enhances hippocampal long-term potentiation (104, 105). These findings are compatible with broader evidence linking lipid mediators to neuroinflammatory modulation and synaptic plasticity.

Given the absence of direct microglial or cytokine profiling, neuroinflammation-associated cognitive dysfunction should be regarded as an exploratory indication, requiring targeted mechanistic validation including assessment of microglial activation states, pro-inflammatory cytokine production, and blood–brain barrier integrity in relevant disease models.

### 5.9 Integrative perspective

Across disease domains, MacDGs display a consistent mechanistic footprint: attenuation of STAT3/STAT6 and NF-κB signaling, normalization of chemokine-driven leukocyte trafficking, and protection of epithelial-rich tissues from inflammatory injury. Chemotherapy-associated mucositis and neutropenia, allergic airway disease, and atopic dermatitis currently represent the most translationally advanced indications. Establishing the primary molecular target(s) and dose–response relationships in well-defined patient populations will determine whether MacDGs advance as niche epithelial protectants or broadly applicable inflammatory modulators.

## 6 Translational barriers and knowledge gaps

The translational advancement of MacDGs remains constrained by substantial uncertainties spanning molecular biology, pharmacology, and clinical development. Although preclinical and early clinical findings suggest a coherent immunomodulatory profile, the evidentiary foundation is narrow, centered on a single synthetic analog, and limited by fundamental unanswered questions.

### 6.1 Fundamental biological uncertainties

No specific acyltransferases, esterases, or remodeling enzymes generating or degrading MacDGs have been identified in mammalian systems, preventing construction of endogenously relevant biochemical pathways. The discovery of MacDGs in mammalian tissues including bovine udder and deer antler, with natural extracts demonstrating hematopoietic stem cell-stimulating activity (28, 31, 41), raises critical questions. Notably, the hematopoietic properties documented for deer antler-derived MacDGs have not been reproduced with synthetic PLAG. Potential explanations include structural divergence between complex natural mixtures and pure synthetic PLAG, presence of bioactive co-constituents, concentration-dependent effects, or tissue-specific activation requirements.

Despite extensive downstream characterization, primary molecular targets remain unidentified. Rapid modulation of STAT3, STAT6, and NF-κB pathways implies membrane-proximal engagement, yet no receptors, scaffolding proteins, or adaptors have been identified as direct binding partners. Target ambiguity limits dose optimization, safety assessment, off-target prediction, and biomarker development.

### 6.2 Pharmacological gaps

Nearly all studies employ PLAG, creating profound structure–activity relationship gaps. The broader MacDG chemical space includes diversity in chain length, unsaturation, stereochemistry, and positional isomerism, yet biological properties of variants remain unexplored. Whether PLAG’s immunomodulatory properties reflect class-wide features or unique analog-specific behavior is unknown.

Critical pharmacokinetic gaps persist: whether MacDGs are absorbed intact, how they are transported and distributed, and whether they undergo enzymatic cleavage remain unknown. The sn-3 acetyl fate is particularly uncertain. Native PLAG bioavailability is limited, necessitating advanced delivery systems such as S-SNEDDS (34, 35). However, long-term formulation stability, manufacturing scalability, and performance across diverse patient populations remain uncharacterized. Preliminary pharmacokinetic studies suggest short plasma half-lives, potentially requiring frequent dosing and raising adherence concerns for chronic disease management.

Validated biomarkers reflecting MacDG target engagement or mechanistic activity are absent.

Downstream readouts like reduced STAT3 or NF-κB signaling are too broad and nonspecific. Without direct molecular targets, target-based biomarkers such as receptor occupancy assays cannot be developed. This forces clinical trials to rely on insensitive, variable clinical endpoints.

### 6.3 Disease-specific, validation, and clinical development uncertainties

Critical disease-specific uncertainties complicate therapeutic positioning. In rheumatoid arthritis, whether bone-protective effects reflect direct osteoclast modulation or cytokine suppression consequences is unresolved, affecting positioning relative to RANKL or JAK inhibitors (44, 106, 107). In tumor models, relative contributions of neutrophil depletion, checkpoint synergy, and direct tumor effects require delineation for rational combination design (78, 79, 84). In neuroinflammation, whether acetylcholinesterase and long-term potentiation effects reflect direct neuronal actions, microglial modulation, or systemic anti-inflammation remains uncertain (104, 105).

Critically, whether identical formulations achieve therapeutic thresholds across mechanistically diverse indications (Th2-driven asthma versus neutrophil-driven acute lung injury versus T-cell–driven rheumatoid arthritis) or require indication-specific optimization is unknown. Without biomarkers predicting response or cost-effectiveness analyses, clinical adoption faces significant barriers even with demonstrated efficacy (108).

Comparative effectiveness data establishing MacDG positioning versus existing therapies are entirely absent. In atopic dermatitis, advantages over JAK inhibitors (which carry infection and malignancy risks) or biologics like dupilumab require demonstration through head-to-head trials (109). In rheumatoid arthritis, unique benefits beyond established disease-modifying antirheumatic drugs, JAK inhibitors, or IL-6 antagonists must be shown. Market positioning relative to crowded therapeutic spaces requires demonstrating clear differentiation through superior efficacy, improved safety profiles, reduced treatment costs, or unique mechanistic advantages that complement existing therapies (110).

Much data originates from few laboratories with minimal independent validation. Although internally consistent, lack of methodological diversity and cross-laboratory confirmation limits generalizability. PLAG synthesis, purity, formulation, or delivery variations could influence outcomes. Absent standardized analytical methods for quantification, purity verification, or metabolic profiling increases variability. Broader lipidomics, immunology, and pharmacology community engagement is essential for validating mechanistic claims and building translational confidence beyond initial investigator groups.

Early clinical studies suggest favorable short-term tolerability (19, 101), but long-term safety remains unknown. As modified triglycerides, MacDGs could incorporate into lipid pools, membranes, or lipoproteins with unanticipated chronic exposure effects. Potential concerns include metabolic perturbations, lipid trafficking alterations, oxidative modifications, or interactions with neutral lipid turnover enzymes. Long-term toxicology studies, assessment of cumulative effects, tissue accumulation potential, and metabolic remodeling consequences are absent from current literature.

Safety considerations extend critically to combination therapy, where MacDGs as multinode modulators may prove most clinically relevant (111). Drug–drug interaction studies are absent. Combined use with JAK inhibitors (overlapping STAT3 suppression mechanisms), corticosteroids (compounding immunosuppression risks), or checkpoint inhibitors (uncharacterized synergy and toxicity) lacks safety data (112). Optimal sequencing strategies, dosing adjustments requirements, and monitoring protocols are undefined. Preclinical checkpoint blockade synergy requires controlled clinical validation with standardized efficacy endpoints and systematic toxicity monitoring before clinical deployment.

Despite Phase 2 oral mucositis completion and ongoing atopic dermatitis evaluation (NCT06487000), significant pathway barriers remain. Optimal patient selection criteria, efficacy endpoints most sensitive to MacDG activity, and dose–response relationships in heterogeneous patient populations are poorly defined. Regulatory positioning is uncertain, classification as disease-modifying agent, adjunctive therapy, or supportive care depends on comparative efficacy data currently lacking. Phase 2 to Phase 3 progression faces absent validated surrogate endpoints, necessitating large clinical outcome trials (113). In chronic inflammatory diseases, demonstrating superiority or non-inferiority to established therapies requires challenging head-to-head trials with adequate power and duration.

Intellectual property landscapes are unclear, whether composition-of-matter patents sufficiently prevent generic competition or method-of-use patents provide adequate market exclusivity affects commercial developer investment decisions (114, 115). Health economic modeling incorporating cost-effectiveness analyses, quality-adjusted life years, and budget impact assessments will be critical for securing reimbursement approval from payers, particularly in competitive therapeutic areas where multiple effective treatments already exist (116).

## 7 Future perspectives

Advancing MacDGs toward clinical relevance requires coordinated investigations resolving biochemical, mechanistic, clinical, and commercial uncertainties across multiple interconnected domains.

### 7.1 Foundational research priorities

Establishing endogenous MacDG status in mammalian systems through stable isotope tracing during inflammatory stimulation, comprehensive tissue-specific lipidomic profiling across health and disease states, and enzymatic characterization of candidate acyltransferases or esterases capable of sn-3 acetylation is an immediate priority. Particular focus should reconcile deer antler-derived MacDG hematopoietic activity with synthetic PLAG absence through comparative structural analysis of natural extracts versus synthetic PLAG and functional validation of fractionated natural extracts to identify bioactive components beyond pure PLAG.

Clarifying primary molecular targets represents a parallel foundational priority. Mechanistic ambiguity limits rational analog design, biomarker identification, and dosing strategies. Approaches integrating proximity labelling with clickable MacDG analogs, affinity-based lipid probes coupled with quantitative proteomics, membrane fractionation identifying protein–lipid complexes, and high-resolution structural methods such as cryo-electron microscopy may reveal interacting receptor complexes, scaffolding proteins, or lipid microdomain components. Complementary biophysical studies examining membrane order, curvature, and lateral organization using fluorescence recovery after photobleaching, atomic force microscopy, and molecular dynamics simulations could define how MacDG incorporation alters signaling-relevant membrane properties and receptor clustering dynamics.

Structure–activity relationship mapping must systematically span diverse MacDG species, variations in fatty acid chain length (C14-C22), degree of saturation (saturated, monounsaturated, polyunsaturated), stereochemistry (sn-1/sn-2 regioisomers), and acetyl group positional isomerism, supported by computational modeling of membrane insertion dynamics and biophysical interaction prediction. Comprehensive SAR understanding would enable lead candidate identification with enhanced potency, improved metabolic stability, and better tissue targeting while clarifying whether PLAG represents an optimal therapeutic structure or merely a convenient first-generation compound requiring medicinal chemistry optimization.

### 7.2 Translational pharmacology priorities

Rigorous pharmacokinetic and metabolic fate studies must resolve fundamental questions regarding absorption (intact versus hydrolyzed), distribution (plasma protein binding, tissue partitioning), metabolism (ester hydrolysis kinetics, fatty acid β-oxidation), excretion routes, and tissue persistence through systematic analyses. Comprehensive metabolite profiling using liquid chromatography–tandem mass spectrometry and nuclear magnetic resonance spectroscopy, assessment of cellular lipid pool and membrane fraction incorporation, and characterization of interactions with lipoprotein and lipid transport pathways are essential. Particular attention to sn-3 acetyl group fate, whether rapidly cleaved by esterases or retained within intact MacDGs, and identification of bioactive metabolites (if acetyl cleavage generates DAG-like species) is critical for mechanistic interpretation.

Formulation science must advance beyond preliminary self-nanoemulsifying systems to optimized delivery platforms addressing bioavailability limitations, reducing dosing frequency, and improving patient compliance. Comparative studies of oral versus parenteral administration routes, systematic food effect and drug–drug interaction evaluations, and development of modified-release formulations for extended dosing intervals are warranted. Stability under physiological pH ranges and storage conditions requires rigorous characterization using accelerated stability protocols, and scalable good manufacturing practice-compatible production processes must be established to support clinical development and eventual commercialization.

Pharmacodynamic biomarker development capable of tracking *in vivo* MacDG activity represents a critical translational milestone enabling dose optimization and mechanistic confirmation. Strategies include quantification of plasma or peripheral blood mononuclear cell MacDG levels and metabolites, ex vivo measurement of membrane biophysical properties in accessible cell populations, transcriptional profiling of STAT-responsive genes in peripheral blood or skin biopsies, and multiplex analysis of serum chemokine networks reflecting immune cell trafficking patterns. Early clinical validation of candidate biomarkers would enable dose optimization, mechanistic hypothesis confirmation, and patient stratification in larger efficacy trials. Integrating lipidomic, transcriptomic, and proteomic platforms may reveal composite signatures predictive of therapeutic response.

### 7.3 Clinical development strategies

Completed oral mucositis Phase 2 results and ongoing atopic dermatitis evaluation (NCT06487000) provide critical learning opportunities for refining patient selection criteria, optimizing dose regimens, and establishing clinically meaningful endpoints (19). *Post-hoc* responder versus non-responder analyses, if feasible with available sample sizes, could reveal predictive biomarkers or patient characteristics informing subsequent trial enrichment strategies.

Comparative effectiveness studies positioning MacDGs relative to existing standard-of-care therapies are essential for regulatory approval and market access. In atopic dermatitis, head-to-head comparisons with JAK inhibitors or active-controlled trials versus dupilumab (anti-IL-4Rα) would establish whether MacDGs offer superior efficacy, improved safety profiles (particularly reduced infection or malignancy risks compared to JAK inhibitors), or complementary mechanisms justifying combination approaches (109, 112). In rheumatoid arthritis, comparisons with methotrexate, JAK inhibitors, or interleukin-6 receptor antagonists would define treatment algorithm positioning and identify patient subgroups most likely to benefit.

Combination therapy strategies leveraging observed synergies with checkpoint inhibitors, disease-modifying agents, or conventional chemotherapies require systematic investigation guided by defined mechanistic hypotheses. In oncology settings, observed anti-PD-1/anti-PD-L1 checkpoint inhibitor enhancement (79, 84, 103) warrants clinical evaluation in biomarker-driven patient populations with high tumor-associated neutrophil burdens or immunosuppressive microenvironments. Optimal sequencing strategies, toxicity-managing dosing adjustments, and safety monitoring frameworks must be established through carefully designed Phase 1b/2 combination studies with embedded pharmacodynamic assessments.

### 7.4 Expanding scientific collaboration

Independent replication by groups beyond initial investigators is essential for establishing scientific credibility and identifying potential confounding factors related to synthesis methods, formulation approaches, or model-specific effects. The research community expansion must include independent academic laboratories, contract research organizations with standardized preclinical platforms, and industrial partners capable of validating mechanistic claims, reproducing key findings across multiple experimental systems, and extending observations to new disease models and therapeutic contexts. Multi-center collaborative networks with standardized protocols, shared reagent repositories ensuring consistent PLAG quality, and coordinated analytical platforms for lipidomic and biomarker measurements would accelerate validation processes.

Cross-disciplinary collaboration among lipid biologists, membrane biophysicists, immunologists, pharmacologists, formulation scientists, and clinicians is essential for integrating mechanistic insights with therapeutic development imperatives. Industry engagement would provide critical resources for large-scale pharmacology studies, good manufacturing practice-compliant production, and pivotal clinical trials beyond typical academic capacity.

Intellectual property landscape clarification is necessary for attracting sustained commercial investment. Freedom-to-operate analyses, evaluation of composition-of-matter versus method-of-use patent strategies, assessment of patent term extension opportunities or orphan drug designation potential will inform development pathways and partnership structures. Commercial viability assessments must realistically consider development costs (including advanced formulation requirements), addressable market sizes across multiple indications, competitive landscape evolution (particularly emerging JAK inhibitors and biologics), and pricing requirements relative to established therapies. Health economic modeling incorporating rigorous cost-effectiveness analyses, quality-adjusted life year calculations, and budget impact projections will be critical for securing favorable reimbursement decisions from payers, particularly in highly competitive therapeutic areas where demonstrating value beyond efficacy becomes essential for market access.

## 8 Conclusion

Monoacetyldiacylglycerols have emerged as intriguing lipid modulators with reproducible effects across inflammatory and injury-related contexts. Their suppression of STAT3, STAT6, and NFκB signaling, recalibration of neutrophil dynamics, promotion of resolution-associated macrophage functions, and protection of epithelial barriers under stress provides coherent mechanistic rationale for evaluation in chronic inflammatory diseases. These properties distinguish MacDGs from classical anti-inflammatory agents by targeting membrane-proximal events and integrating effects across immune and epithelial compartments. The breadth of preclinical activity, combined with preliminary clinical findings in chemotherapy-associated mucositis and neutropenia, underscores potential translational importance.

However, the field is defined as much by unknowns as by established findings. Absent validated molecular targets, unclear endogenous biology, and incomplete pharmacokinetic understanding represent substantial clinical development barriers. Reliance on a single synthetic analog limits generalizability, while scarcity of independent replications highlights the need for broader scientific engagement.

Nonetheless, mechanistic coherence across experimental systems, coupled with compatibility with fundamental cellular processes, suggests this lipid class may represent a novel immunomodulatory frontier. Their capacity to modulate multiple upstream inflammatory circuits simultaneously aligns with chronic inflammatory disease complexity and may complement existing cytokine- or receptor-targeted therapies. Coordinated efforts in structural biology, lipidomics, mechanistic immunology, and translational pharmacology will determine whether MacDGs fulfil their promise as multinode lipid regulators of inflammation.
